# Idiopathic acute transverse myelitis: outcome and conversion to multiple sclerosis in a large series

**DOI:** 10.1186/1471-2377-13-135

**Published:** 2013-10-03

**Authors:** Álvaro Cobo Calvo, M Alba Mañé Martínez, Agustí Alentorn-Palau, Jordi Bruna Escuer, Lucía Romero Pinel, Sergio Martínez-Yélamos

**Affiliations:** 1 Multiple Sclerosis Unit, Neurology Department, Hospital Universitari de Bellvitge – IDIBELL, Feixa Llarga s/n L’Hospitalet de Llobregat, Barcelona 08907, Spain; 2 Service de neurologie, Groupe hospitalier Pitié-Salpêtrière, 47-83 boulevard de l’Hôpital, 75651, Paris cedex 13, France; 3 Neuro-oncology Unit, Neurology Department, Hospital Universitari de Bellvitge – IDIBELL, Feixa Llarga s/n L’Hospitalet de Llobregat, Barcelona 08907, Spain

**Keywords:** Transverse myelitis, Idiopathic, Multiple sclerosis, Prognosis, Neuromyelitis optica

## Abstract

**Background:**

In 2002, the Transverse Myelitis Consortium Working Group (TMCWG) proposed the diagnostic criteria for idiopathic acute transverse myelitis (IATM) to delimit and unify this group of patients. This study aimed to describe the conversion rate to multiple sclerosis (MS) and variables associated with conversion, and to analyze functional outcome and prognostic factors associated with functional recovery in patients who fulfilled the current TMCWG criteria for definite and possible IATM.

**Methods:**

Eighty-seven patients diagnosed with IATM between 1989 and 2011 were retrospectively reviewed. Two patients with positive neuromyelitis optica IgG serum antibodies were excluded. Epidemiological, clinical, laboratory, magnetic resonance imaging (MRI) data and outcome of 85 patients were analyzed.

**Results:**

Eleven (13%) patients converted to MS after a median follow-up of 2.9 years (interquartile range 1.0-4.8). Early-age onset of symptoms was related to conversion to MS. Only 9.4% of patients with IATM were unable to walk unassisted at the end of follow-up. Urinary sphincter dysfunction (odds ratio [OR] 3.37, 95% confidence interval [CI] 1.04-10.92) and longitudinally extensive transverse myelitis (LETM) on MRI (OR 12.34, 95% CI 3.38-45.00) were associated with a poorer outcome (Rankin ≥ 2).

**Conclusions:**

At least 13% of patients who fulfill the TMCWG criteria for definite and possible IATM will convert to MS. Functional recovery in IATM is poorer in patients with urinary sphincter dysfunction at admission or LETM on MRI.

## Background

In 2002, the Transverse Myelitis Consortium Working Group (TMCWG) presented the proposed diagnostic criteria for idiopathic acute transverse myelitis (IATM) to delimit and unify this group of patients [1]. Since that time, identification of new diagnostic biomarkers, such as neuromyelitis optica (NMO) IgG antibodies has contributed to differentiate new conditions from the idiopathic group of acute transverse myelitis [2].

Another distinctive group of patients with an IATM event meeting the TMCWG criteria are individuals who present with a first attack of multiple sclerosis (MS) in the setting of an initially normal brain magnetic resonance imaging (MRI) study. The percentage of patients with TMCWG criteria for IATM who have a first demyelinating MS event remains to be clarified.

Individuals considered at a high risk of disability after a first IATM event may benefit from treatments that are more aggressive, such as plasma exchange. Hence, there is a need to identify this cohort of patients early in the course of the disease.

The aim of this study was 1) to describe the conversion rate to MS and identify variables associated with conversion, and 2) to analyze the functional outcome and prognostic factors associated with recovery in patients who fulfill the current TMCWG criteria for possible and definite IATM.

## Methods

### Patients

The study was approved by the Clinical Research Ethics Committee of Bellvitge University Hospital and all data were collected in an anonymized fashion (ref PR228/12).

We reviewed 604 medical records from patients with neurological signs attributed to spinal cord inflammation assessed at Bellvitge University Hospital (Barcelona, Spain) between January 1989 and December 2011. Among them, we found 487 patients diagnosed with MS who presented a spinal cord inflammation or a first spinal cord inflammation with cranial MRI suggestive of MS, 11 due to parainfeccious disease, systemic disease in 9, spinal cord infarction in 3 patients and ADEM, dural fistula and tumoral-related spinal cord in 2 patients respectively. One patient had simultaneous myelitis and optic neuritis (ON) as a part of the classic Devic Syndrome. We found out 87 patients initially diagnosed with IATM and no history of previous neurological symptoms.

We then retrospectively studied these 87 patients. Patients who fulfilled the criteria for IATM proposed by the TMCWG (definite IATM) and those who met all criteria with the exception of 'objective documentation of inflammation within the spinal cord’ (possible IATM) were included and analyzed in the present study. The current cohort includes the patients who were described in a previously published study [3].

In 38 of the 87 patients, serum samples were tested for NMO-IgG antibodies by immunohistochemistry, as described elsewhere [4]. NMO-IgG antibodies could not be systematically sought, as the technique became available in our hospital in 2007. This validated technique has shown a sensitivity (S) of 50-60% and a specificity (Sp) of more than 90%. Two patients showed positive reactivity and were excluded from the study. Both patients developed NMO disease, based on the diagnostic criteria for this condition [5].

The 85 patients fulfilling the criteria for definite and possible IATM were classified into two groups: the IATM group and the MS group. The diagnosis of MS was based on the revised Poser or McDonald criteria [6,7].

### Epidemiological and clinical data

Sex, age at symptoms onset, season of onset and time to reach maximum functional deficit were recorded. Hypertension, hypercholesterolemia, diabetes mellitus, smoking habit, traumatic events, and symptoms related to infection (eg, flu, gastrointestinal, or urinary tract symptoms) within one month before the onset of IATM symptoms were also collected. Clinical findings were noted, such as urinary sphincter dysfunction or anal sphincter dysfunction, and the treatment received. None of the patients were receiving immunosuppressants or corticosteroids before the acute spinal event or at the time immunological studies were performed.

Disability was evaluated on admission and at last visit using the modified Rankin Scale [8]. Rankin scores were categorized into <2 and ≥2 to distinguish functional disability. Patients who converted to MS were further evaluated by the Expanded Disability Status Scale (EDSS) to determine the extent of the neurological disability at the last visit.

### Laboratory data

A spinal tap was performed in all patients before treatment. CSF was analyzed for glucose concentration, cell count, and IgG index (positive, >0.7), and immunofixation electrophoresis was used for oligoclonal band (OCB) testing.

### Imaging data

In all patients, baseline and follow-up MRI scans were performed on a 1.5-Tesla system. MRI study included axial and sagittal images of the brain and spinal cord obtained by T1, T2, FLAIR and T1 post-contrast sequences. All patients had normal brain MRI findings at the first episode of IATM. All underwent spinal MRI on admission, and the number, location, and length of lesions were noted. Longitudinally extensive transverse myelitis (LETM) was defined as extension over three or more vertebral segments on MRI study.

### Follow-up

Patients were followed-up with a neurological evaluation every 6 months during the first year, and once a year thereafter. Follow-up time was established as the difference between the date of the last visit and the date of admission. Analysis of data was performed on December 2012. An additional neurological examination was performed when a relapse occurred. Relapse was defined as a second demyelinating event at the spinal cord (recurrence) or other central nervous system structure at least one month after the first event, sustained for a minimum of 24 hours. The date of MS conversion was noted in all patients in the MS group.

### Statistical analysis

Categorical variables and ordinal variables were compared with the chi-square test and Mann–Whitney *U* test, respectively. Correlations between ordinal variables were analyzed with Pearson’s correlation coefficient. Quantitative continuous variables were compared with the Student *t* test. Multivariate logistic regression analysis was performed using the forward stepwise method and including only those variables that were statistically different in the univariate analysis at a significance level of *p-*value < 0.05. After the Bonferroni correction the level of significance for multiple comparison was established at *p-*value < 0.001.

## Results

### Epidemiological and clinical data

Among the 85 IATM patients included in the study, 45 (53%) were women and mean age at onset was 43 ± 16.2 years. Seventy-four patients were ultimately classified in the IATM group (87%) and 11 (13%) in the MS group. Epidemiological and clinical data are shown in Table 1 and Table 2.

**Table 1:** Epidemiological and clinical characteristics of patients with low versus high Rankin Scores at the final of the follow up

	**Outcome Rankin**		***p***	**Total**
	**<2**	**≥2**		
N (%)	51 (60)	34 (40)		85
Age, mean (SD), y	38.9 (14)	49 (17.7)	*0.005*	43 (16.2)
Sex, male/female	21/30	19/15	*0.194*	40/45
Follow up, median (IQR), y	2.9 (1.5-4.1)	2.7 (0.3-7.4)	*0.683*	2.9 (1–4.8)
Time to maximum deficit, mean (SD), d	7.2 (7)	6.91 (7.6)	*0.821*	7.1 (7.1)
Admission Rankin Mean (SD)	2.27 (1)	3.65 (1.1)	*<0.001*^*a*^	2.82 (1.2)
Outcome Rankin Mean (SD)	____	____	*____*	1.4 (1.3)
Time onset-start treatment, mean (SD), d	9.7 (8.3)	8.5 (8)	*0.602*	9.2 (8.1)
Back pain, n (%)	19 (37.3)	16 (47.1)	*0.380*	35 (41.2)
Urinary sphincter dysfunction, n (%)	21 (41.2)	26 (76.5)	*0.001*^*a*^	47 (55.3)
Anal sphincter dysfunction, n (%)	2 (4)	9 (26.5)	*0.002*	11 (13)
Previous infection, n (%)	19 (37.3)	8 (23.5)	*0.237*	27 (31.8)
Summer season/Not summer season, n (%)	9/49 (18) /40/49 (81.6)	5/34 (14.7) / 29/34 (85.3)	*0.661*	14 (16.47) /69 (81.17)
TM relapses, n (%)	5/51 (9.8)	0/33 (0)	*0.085*	5/84 (6)
MS conversion, n (%)	8 (15.7)	3 (8.8)	*0.513*	11 (13)

*IQR:* Interquartile range.

*SD:* Standard deviation.

*TM:* Transverse myelitis.

^*a*^Significant after Bonferroni correction.

**Table 2:** Epidemiological and clinical characteristics comparing IATM versus MS group

	**IATM**	**MS**	***p***
	**group**	**group**	
N (%)	74 (87)	11 (13)	
Age, mean (SD), y	45 (16)	28.2 (9.1)	*0.001*^*a*^
Sex, male/female	36/38	4/7	*0.529*
Follow up, median (IQR), y	2.9 (0.9–3.9)	9.9 (2.7–17.8)	*<0.001*^*a*^
Time to maximum deficit, mean (SD), d	6.9 (7.2)	8.5 (7)	*0.487*
Admission Rankin, Mean (SD)	2.8 (1.2)	2.45 (1.1)	*0.284*
Outcome Rankin Mean (SD)	1.3 (1.3)	1.8 (1.2)	*0.290*
Time onset-start treatment, mean (SD), d	9 (8.1)	2 (2.2)	*0.553*
Back pain, n (%)	30 (40.5)	5 (45.5)	*0.755*
Urinary sphincter dysfunction, n (%)	41 (55.4)	6 (54.5)	*0.603*
Anal sphincter dysfunction, n (%)	10 (13.5)	1 (9.1)	*0.566*
Previous infection, n (%)	23 (31.1)	4 (36.4)	*0.485*
Summer season/Not summer season, n (%)	13/72(18.1)/59/72 (81.9)	1/11 (9.1)/10/11 (90.9)	*0.460*
TM relapses, n (%)	0/73 (0)	5/11 (45.5)	*<0.001*^*a*^

*IATM:* Idiopathic acute transverse myelitis.

*MS:* Multiple Sclerosis.

*IQR*: Interquartile range.

*SD:* Standard deviation.

*TM:* Transverse myelitis.

^*a*^Significant after Bonferroni correction.

In the MS group, patients with a relapse after the first spinal cord event presented with typical symptoms of MS; three had ON, one sensory and sphincter dysfunction, one sensory and motor symptoms, two pure motor symptoms and three pure sensory symptoms. Nine patients had relapsing-remitting MS and two progressed to secondary progressive MS (SPMS). One SPMS patient experienced no further relapses after the first spinal attack.

At the end of follow-up, myelitis recurred in 5 patients, all in the MS group. No patients with LETM had recurrences. Two patients with a first LETM who converted to MS did so at one and seven months from the first attack, respectively.

Patients with onset of symptoms in summer reached nadir faster than the remaining patients (7.94 days, SD 7.5 *vs* 3.14 days, SD 3.08*, p* < 0.001*)*.

Treatment with intravenous methylprednisolone (1 g daily for 5 days) was given to 67 patients. Three patients (two of whom presented as LETM) needed further treatment, such as plasma exchange, due to a poor response to corticosteroids and symptoms of impairment at admission (Rankin Score was 3, 5 and 4, respectively).

Among the MS group, seven patients were treated with disease modifying drugs during the follow up.

### CSF findings

CSF cell count was determined in 77 patients and pleocytosis (total cellularity >5 cell/mm^3^) was found in 18 (23.4%). Among the 10 MS group patients in whom cell count was performed, 5 had less than 5 cell/mm^3^ and only 1 had more than 50 cell/mm^3^.

Five out of 15 patients with OCB in CSF developed MS compared with only one out of 44 without OCB. The presence of OCB was associated with conversion to MS, but the results were not statistically significant after Bonferroni adjustment (*p* = 0.003) (S 83.3%, Sp 81.5%, positive predictive value [PPV] 33.3%, negative predictive value [NPV] 97.8%). The IgG index was not, however, related to MS conversion (*p* = 0.800) (S 60%, Sp 80%, PPV 23.1%, NPV 95%). The combination of positive OCB testing and IgG index >0.7 yielded a negative predictive value and sensitivity of 100% to MS conversion. (S 100%, Sp 68.6%, PPV 23.8%, NPV 100%) (*p* = 0.011). CSF data are shown in Table 3 and Table 4.

**Table 3:** CSF and MRI characteristics with low versus high Rankin Scores at the final of the follow up

	**Outcome Rankin**		***p***	**Total**
	**<2**	**≥2**		
N (%)	51 (60)	34 (40)		85 (100)
*CSF (mean, SD)*				
Glucose, mmol/L	3.5 (1.1)	4 (1)	*0.035*	3.7 (n:78)
Cell count, n/mm^3^	8.9 (24.1)	27.8 (125.4)	*0.321*	16.5 (n:77)
Protein, g/L	0.4 (0.2)	0.5 (0.2)	*0.389*	0.5 (n:78)
OCB+, n (%)	12/40 (30)	3/20 (15)	*0.343*	15/60 (25)
IgG index >0.7, n (%)	8/33 (24.2)	5 /22 (22.7)	*0.581*	13/ 55 (23.6)
*MRI*				
Vertebral segments, n, mean (SD)	1.7 (1.9)	3.8 (3.3)	*0.003*	2.5 (2.8)
LETM (%)	(9.8)	21 (61.8)	*<0.001*^*a*^	26 (30.6)
GD+, n (%)	14/30 (46.6)	8/21 (38.1)	*0.800*	22/51 (43.1)
Time onset-MRI, Mean (SD), d	9.84 (7.2)	8.37 (7.5)	*0.408*	9.26 (7.3)

*CSF:* Cerebrospinal fluid.

*OCB+:* Positive oligoclonal band test.

*MRI: M*agnetic resonance imaging.

*LETM:* Longitudinally extensive transverse myelitis.

*GD+:* Gadolinium enhancement.

*SD*: standard deviation.

^a^Significant after Bonferroni correction.

**Table 4:** CSF and MRI characteristics comparing IATM versus MS group

	**IATM**	**MS**	***p***
	**group**	**group**	
N (%)	74 (87)	11 (13)	
*CSF (mean, SD)*			
Glucose, mmol/L	3.7 (1.1)	3.4 (0.6	*0.424*
Cell count, n/mm^3^	17.4 (87.1)	10 (18.4)	*0.789*
Protein, g/L	0.5 (0.2)	0.35 (0.2)	*0.044*
OCB+, n (%)	10/54(18.5)	5/6 (83.3)	*0.003*
IgG index >0.7, n (%)	10/50 (20)	3/5 (60)	*0.800*
*MRI*			
Vertebral segments, n, mean (SD)	2.6 (2.8)	2 (2.2)	*0.403*
LETM (%)	24 (32.4)	2 (18.2)	*0.281*
GD+, n (%)	20/43 (46.5)	2/8 (25)	*0.309*
Time onset-MRI, Mean (SD), d	8.56 (7)	14.22 (8.1)	*0.029*

*IATM:* Idiopathic acute transverse myelitis.

*MS:* Multiple Sclerosis.

*MRI:* Magnetic resonance imaging.

*CSF:* Cerebrospinal fluid.

*OCB+:* Positive oligoclonal band test.

*LETM:* Longitudinally extensive transverse myelitis.

*GD+:* Gadolinium enhancement.

*SD:* Standard deviation.

^a^Significant after Bonferroni correction.

### MRI findings

Spinal cord MRI revealed a cervical lesion in 31 (36.5%), thoracic lesion in 33 (38.8%), and lumbar lesion in 11 (12.9%) of 85 patients. In 19 (22.5%) patients, no abnormalities were observed in the first spinal cord MRI. Five of these 19 patients showed abnormal CSF findings (positive IgG index and/or pleocytosis) and one patient had a cervical lesion in the second spinal cord MRI. The remainder showed no abnormal findings in the second MRI. All the patients with a normal first spinal cord MRI had abnormal neurological signs suggestive of transverse myelitis, as the TMCWG points out. There were no statistically significant differences between the IATM and MS groups with regard to the location of the MRI signal abnormality. None of the patients in the MS group developed lumbar myelitis.

Within the MS group, six patients fulfilled the Barkhof criteria in a subsequent brain MRI. The distributions of the lesions in the axial plane at the first spinal MRI were predominantly posterior (4/11) and lateral (2/11), and two patients had a lesion area that extended over more than 50% of the spinal cord section. In three MS patients, the first spinal MRI was normal and the subsequent spinal study revealed a single cervical lesion in one of them. Nine MS patients underwent a second spinal MRI; in three the lesion had resolved, three had new spinal lesions that extended over fewer than three vertebral segments, and three showed no changes.

LETM was observed in 26 (30.6%) patients and was related to greater disability. In this group, 81% of patients had a Rankin score ≥2 at their last visit compared with 22% of patients without LETM (*p* < 0.001). The fact of having LETM did not rule out conversion to MS: two of 26 patients (7.5%) with LETM developed MS.

Twenty-two of 26 patients with LETM presenting as IATM were seronegative for NMO-IgG antibodies at presentation and during follow-up. In the four remaining LETM patients, NMO-IgG antibodies were not analyzed: two patients converted to MS and two died from myelopathy-unrelated etiology after two and three years of follow-up, respectively. Additional MRI data are shown in Table 3 and Table 4.

### Follow-up and outcome

**Figure 1:**
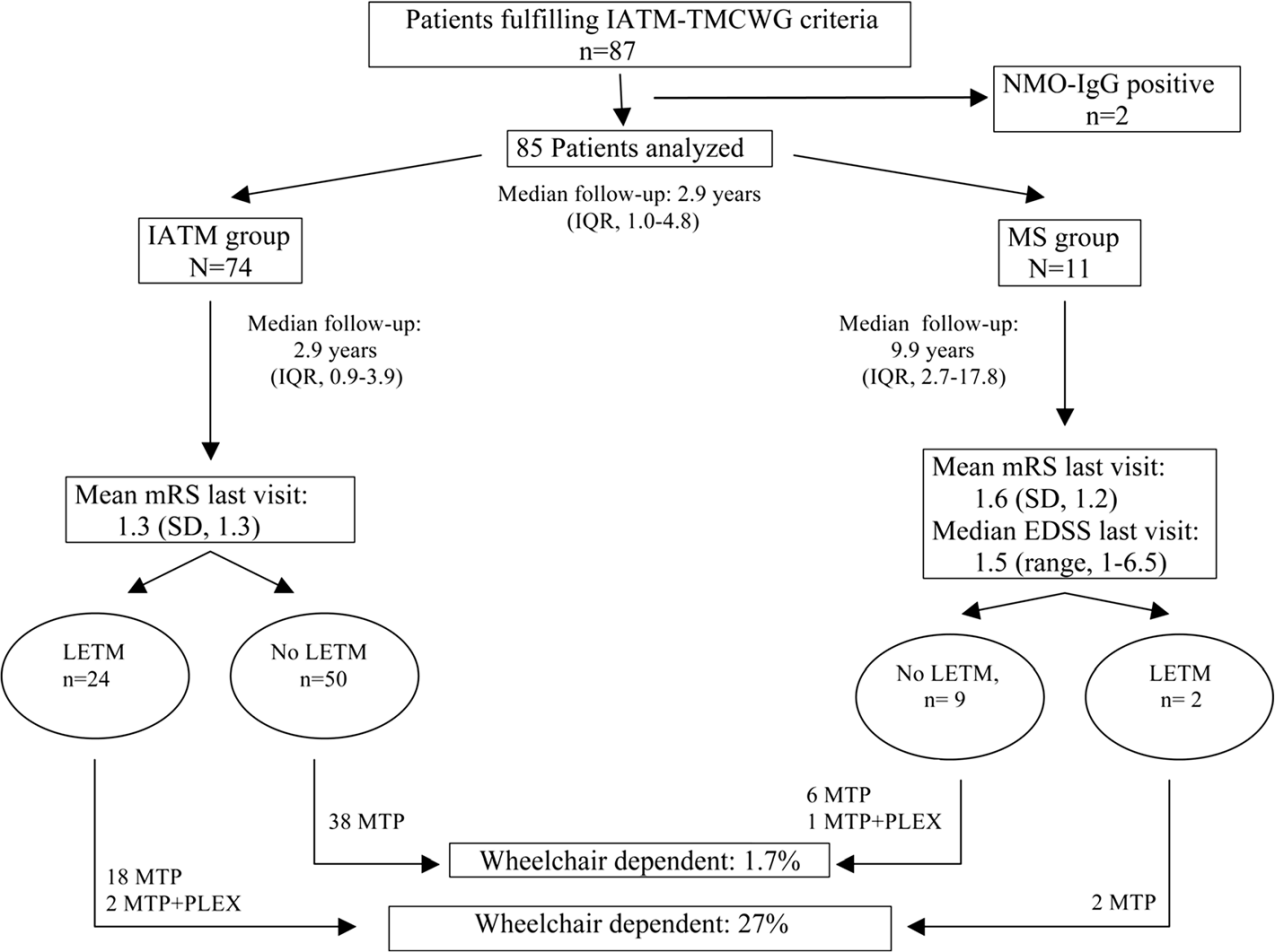
**Diagram reflecting the prognosis of outcome in the different subgroups. ***IATM* idiopathic acute transverse myelitis, *TMCWG* Transverse Myelitis Consortium Working Group, *LETM* longitudinally extensive transverse myelitis, *NMO-IgG* neuromyelitis optica IgG antibodies, *MTP* methylprednisolone, *PLEX*, plasma exchange, *EDSS* Expanded disability Status Scale, *mRS* modified Rankin Scale, *IQR* interquartile range.

Among the whole cohort, the median follow-up time was 2.9 years (IQR 1.0-4.8). The median follow- up was quite long in the MS group, which had a median follow- up time of 9.9 years (IQR 2.7-17.8) compared to the IATM group with a median of 2.9 years (IQR 0.9-3.9). The patients converted to MS at a median of 1.2 years (IQR 0.37-1.87) after the first spinal cord event. At completion of follow-up, early age at onset of symptoms was related to conversion to MS in patients who fulfilled criteria for possible or definite IATM (Table 2). Data related to follow-up and outcome are shown in Figure 1.

Modified Rankin Score results at admission and end follow-up are shown in Table 5. A higher admission Rankin score was significantly related to a poorer outcome (Pearson correlation coefficient 0.65; *p* < 0.001). Thirty-three patients (97.1%) with functional disability at the last visit had an admission Rankin ≥2 (*p* = 0.006). Among 71 patients with a score ≥2 at admission, 33 (46.5%) had an outcome Rankin ≥2 (*p* = 0.006).

**Table 5:** Disability at admission and last follow-up visit

**mRS**	**Admission**	**Last visit**
	**n (%)**	**n (%)**
0	0	27 (31.8)
1	14 (16.5)	26 (30.6)
2	22 (25.9)	11 (12.9)
3	21 (24.7)	13 (15.3)
4	21 (24.7)	7 (8.2)
5	7 (8.2)	1 (1.2)
6	0	0

*mRS* modified Rankin Scale.

Urinary sphincter dysfunction was the only clinical symptom related to Rankin ≥2 at last visit (*p* < 0.001) (Table 1).

Independent risk factors associated with an outcome Rankin score ≥2 were urinary sphincter dysfunction (OR 3.37, 95% CI 1.04-10.92) and LETM (OR 12.34, 95% CI 3.38-45.00).

## Discussion and conclusions

To the best of our knowledge, the 85 patients in the present study represent the largest series meeting the criteria for possible and definite IATM established by the TMCWG.

NMO spectrum disorders (NMOSD) are considered limited forms of NMO, such as single or recurrent LETM events or relapsing or simultaneous bilateral ON with positive serum NMO-IgG testing [9]. At onset of symptoms, NMO may not be easily distinguishable from IATM presenting as LETM, and NMO-IgG has been suggested as the most reliable marker to identify patients with this condition [2]. However, a recent report has indicated that positive NMO-IgG status may not be needed to reach a definite diagnosis in 90% of patients with clinically suspected NMO [10]. Among the idiopathic LETM patients presented here, all were seronegative, although in four patients, NMO-IgG were not analyzed. Two of these patients converted to MS and the other two died without presenting further relapses of either myelitis or ON. After excluding NMOSD and MS, recurrence of IATM or even LETM is rare in our experience. Monophasic isolated LETM might represent a distinct clinical syndrome, as others have suggested [11,12].

Studies analyzing CSF in IATM have reported pleocytosis in a high percentage of patients (42%-62%) [13-15]. Because patients with spinal cord ischemia show no CSF pleocytosis, it has been proposed that CSF leukocyte count could be a useful marker to differentiate inflammatory myelopathies from spinal cord infarcts [16]. In our series, however, a lower percentage of patients had pleocytosis (23.4%) than the reported rates. Because normal CSF cell counts can be found in both conditions, this marker would likely be of limited usefulness to identify IATM.

According to our findings, IATM presenting as LETM does not rule out a first demyelinating MS. These results are in agreement with a prospective study investigating LETM in white patients [17]. LETM has been associated with a poorer prognosis in patients with acute transverse myelitis [18,19] and recently, longer extension of LETM has been related to a higher number of relapses [17]. In our series, LETM was the most important independent risk factor for long-term disability.

Up to 50% of patients with neurological symptoms suggestive of spinal cord inflammation had normal spinal cord MRI [20]. In our study, 15.2% of patients neither showed evidence of lesions at first or subsequent spinal MRI nor revealed paraclinical data supporting inflammation within spinal cord. TMCWG criteria includes such a patients as 'possible ATM’.

Early studies reported that up to one third of patients with a first episode of acute transverse myelitis remained unable to walk [21,22]. Most studies carried out after establishment of the TMCWG criteria disclosed similar rates of poor outcome ranging from 11% to 35.8% [13,18,23]. In our series, only 9.4% of patients were unable to walk unassisted at completion of follow-up (Rankin ≥4). Differences in the length of follow-up, together with methodological aspects (collaborative multicenter studies *vs* hospital-based series), may have contributed to the better outcome in our cohort, compared to others [14,17].

Several clinical factors associated with a poor outcome have been described in patients with acute transverse myelitis, including back pain, severe functional deficit and motor involvement at onset of symptoms, symptoms progression within 24 hours, relapse occurrence, and spinal shock [3,14,21,22,24]. As was mentioned above, among the para-clinical factors, LETM has been associated with a poor prognosis in these patients [18,19]. The multivariate analysis in the present study showed that urinary sphincter dysfunction and LETM were independently associated with a poor prognosis in IATM.

Regarding patients with a first event involving an isolated spinal cord lesion, it is well recognized that partial myelitis is highly predictable of progression to MS [15,25]. Previous studies in IATM have reported a conversion rate of 0% [14,18] to 11.4% [23], with a mean follow-up ranging from 2 to 4.8 years. Therefore, in accordance with the results of our study, conversion to MS may occur even after an appropriate follow-up of IATM patients. MS patients had a longer median follow-up compared with IATM patients. Although this difference might result in a lower conversion rate in the IATM group, MS patients showed a median time to conversion of 1.2 years (IQR 0.37-1.87) and the median follow-up time in the IATM group was 2.9 years. Therefore, IATM patients will unlikely convert to MS.

IATM patients testing positive for OCB in CSF had an increased risk of developing MS in the follow-up (33.3% vs 2% conversion rate). In contrast, the combination of negative OCB testing and IgG index ≤0.7 yielded a very low likelihood of converting to MS (NPV 100%). These data are in keeping with the results of previous studies investigating the utility of CSF findings in patients with a clinically isolated syndrome who convert to MS [26].

Age, family background, sensory symptoms, greater disability at onset of symptoms, brain MRI lesions, presence of OCB, and an abnormal CSF IgG index are reported predictive factors of conversion to MS in patients with a clinically isolated cord syndrome [27-29]. We found that in patients with an initial IATM, onset of symptoms at an early age is related to conversion to MS. In addition, patients with a normal IgG index and no evidence of OCB in CSF have a very low risk of converting to MS.

In addition to the retrospective nature of the case series, our study has other limitations. Firstly, our study was not designed to evaluate treatment response and therefore we could not consider therapeutic aspects when analyzing long term outcome. Secondly, a single centre design may be considered as a limitation although our hospital is a reference centre for patients with clinical symptoms suggestive of acute myelopathy in our sanitary district. Finally, we consider that the patients included herein will unlikely develop NMO: the seronegative status in LETM patients, the absence of other neurological signs such as ON, the relatively good final outcome and the absence of recurrence after almost 3 years of follow-up support the diagnosis of IATM [30,31].

In conclusion, our results indicate that at least 13% of patients who fulfill the TMCWG criteria for definite and possible IATM will convert to MS. Functional recovery in IATM is poorer in patients with urinary sphincter dysfunction at admission and findings of LETM on MRI study.

### Consent

Written informed consent was obtained from all patients.

## Abbreviations

TMCWG: Transverse myelitis consortium working group; IATM: Idiopathic acute transverse myelitis; NMO: Neuromyelitis optica; MS: Multiple sclerosis; MRI: Magnetic resonance imaging; ON: Optic neuritis; S: Sensitivity; Sp: Specificity; EDSS: Expanded disability status scale; CSF: Cerebrospinal fluid; OCB: Oligoclonal band; LETM: Longitudinal extensive transverse myelitis; SPMS: Secondary progressive multiple sclerosis; PPV: Positive predictive value; NPV: Negative predictive value; NMOSD: Neuromyelitis optica spectrum disorders.

## Competing interest

Dr. Martínez Yélamos and Dra. Romero Pinel have received honoraria compensation for participating in AdvisoryBoards, acting as consultant and for speaking activities from Bayer Schering, Biogen idec, Merck-Serono and Teva.

## Authors’ contributions

AC designed the study, collected the data, participated in the statistical analysis and wrote the manuscript. AM and AA contributed to data collection and assisted with the writing manuscript. JB, LR and SMY participated in the design of the study, carry out the statistical analysis and reviewed the manuscript. All authors read and approved the final manuscript.

## Pre-publication history

The pre-publication history for this paper can be accessed here:

http://www.biomedcentral.com/1471-2377/13/135/prepub
